# A High Voltage Current Will Stop a Threatened Alveolar Abscess

**Published:** 1897-03

**Authors:** William Rollins

**Affiliations:** Boston


### A HIGH VOLTAGE CURRENT WILL STOP A THREATENED ALVEOLAR ABSCESS.

BY WILLIAM ROLLINS, BOSTON.

THE generator used was described in this journal for October, 1896, when it was recommended to dentists for use in benumbing the dentine. Electricians differ in regard to the voltage, one placing it at fifty-seven thousand, another at two hundred and fifty thousand. The ampèrage is equally indefinite. When tested before use it ran at a speed of fourteen hundred revolutions, and gave an apparently continuous stream through three-quarters of an inch of air. The patient held the negative terminal in his hand, the positive, covered with cotton, against the most painful place on the palate, which was opposite the apex of the root of the first molar. The feeling of intense cerebral congestion stopped in fifteen minutes; the severe pain in an hour. The gum which beat visibly like a heart became nearly normal. There was no return of the symptoms. In using high voltage currents the following method will prevent the patient from feeling the least sensation from the strongest current which the generator mentioned will give. In the armature circuit place a switch controlled by one of the feet. Have the current turned on the field. Have the patient hold both terminals in the proper positions. Then start the generator by closing the switch. When the sitting is finished open the switch before the patient drops the terminals.

